# High PINCH1 Expression in Human Laryngeal Carcinoma Associates with Poor Prognosis

**DOI:** 10.1155/2018/2989635

**Published:** 2018-03-20

**Authors:** Georgios Tsinias, Sofia Nikou, Theodoros Papadas, Panagiotis Pitsos, Helen Papadaki, Vasiliki Bravou

**Affiliations:** ^1^Department of Otolaryngology, Head and Neck Surgery, University Hospital of Patras, 26504 Patras, Greece; ^2^Department of Anatomy, Medical School of Patras, 26504 Patras, Greece

## Abstract

Focal adhesion signaling to actin cytoskeleton is critically implicated in cell migration and cancer invasion and metastasis. Actin-binding proteins cofilin and N-WASP regulate actin filament turnover, and focal adhesion proteins parvins and PINCH mediate integrin signaling to the actin cytoskeleton. Altered expression of these proteins has been implicated in human cancer. This study addresses their expression and prognostic significance in human laryngeal carcinoma. Protein expressions of cofilin, N-WASP, *α*-parvin, *β*-parvin, and PINCH1 were examined by immunohistochemistry in 72 human laryngeal squamous cell carcinomas. Correlations with clinicopathological data and survival were evaluated. All proteins examined were overexpressed in human laryngeal carcinomas compared to adjacent nonneoplastic epithelium. High expression of PINCH1 was associated significantly with high grade, lymph node-positive, and advanced stage disease. Moreover, high PINCH1 expression significantly associated with poor overall and disease-free survival and high cytoplasmic PINCH1 expression was shown by multivariate analysis to independently predict poor overall survival. In conclusion, we provide novel evidence that focal adhesion signaling to actin cytoskeleton is implicated in human laryngeal carcinogenesis and PINCH1 has prognostic significance in the disease.

## 1. Introduction

Laryngeal cancer is the second most common neoplasm of the upper aerodigestive tract [1]. The majority of the cases (85%–95%) are classified as squamous cell carcinoma. The 5-year survival for laryngeal squamous cell carcinoma is 60%, and despite the progress in the diagnosis and treatment, survival has not improved much over the years [1]. Since invasion and metastasis account for increased morbidity and mortality, understanding the molecular mechanisms underlying these processes and identifying novel biomarkers could lead to the development of more efficient therapeutic approaches.

Cell migration is crucial in tumor invasion and metastasis and is regulated through actin cytoskeleton reorganization and focal adhesion turnover. Actin reorganization leads to the formation of protrusive structures at the leading edge of cancer cells that mediate migration and invasion through the extracellular matrix (ECM) [2]. Cofilin is an actin-binding protein that regulates actin cytoskeleton dynamics and induces the formation of lamellipodia and invadopodia [1–6]. Cofilin severs actin filaments directly creating new barbed ends and also depolymerises old actin filaments providing free actin monomers available for the next cycle of polymerisation [1–6]. Several studies suggest a significant role of cofilin in cancer cell invasion and metastasis [2, 5, 6]. Increased expression of cofilin has been previously demonstrated in various human cancers including nonsmall cell lung cancer and squamous esophageal carcinoma, and overexpression of cofilin was frequently associated with adverse prognosis and resistance to therapy [7–10].

N-WASP (neural Wiskott-Aldrich syndrome protein) another actin-binding protein is also critically involved in the regulation of actin cytoskeleton dynamics [11]. When activated, N-WASP interacts with the Arp2/3 complex resulting in vigorous actin polymerisation at the leading edge of motile cells and in the formation of filopodia and invadopodia [11]. Several studies implicate N-WASP in cancer progression. Colocalization of N-WASP and Arp-2/3 has been demonstrated in the invadopodia of aggressive cancer cell lines, and high expression of N-WASP is associated with cancer metastasis [6, 12–15]. Elevated expression of N-WASP and association with aggressive tumor features and reduced survival have been demonstrated in several human malignancies [9, 13–15].

Focal adhesion proteins *α*-parvin (ILKBP/actopaxin), *β*-parvin (affixin), and particularly interesting new cysteine-histidine-rich protein (PINCH) provide a link between the ECM and the actin cytoskeleton at integrin adhesion sites [16–25]. Parvins and PINCH bind to integrin-linked kinase (ILK) forming the heterotrimeric ILK-parvin-PINCH (IPP) complex that functions as a protein scaffold and signaling hub with a key role in focal adhesion assembly and integrin signaling [22–24]. Apart from the IPP, parvins and PINCH are engaged in multiple protein interactions and through these adaptor functions they promote actin-cytoskeleton remodeling, cell spreading, and cell migration [16–22]. Parvins and PINCH also regulate cell survival in part through effects on ILK and Akt signaling [20–25]. Increased expression of parvins and PINCH has been reported in human cancer promoting cancer cell migration, invasion, and apoptosis resistance [26–31].

Overall, cofilin, N-WASP, and focal adhesion proteins parvins and PINCH regulate cellular processes critically implicated in cancer progression. However, no previous studies, to the best of our knowledge, have addressed their role in human laryngeal cancer. This study aims to evaluate their expression and prognostic significance in human laryngeal squamous cell carcinoma.

## 2. Materials and Methods

### 2.1. Tissue Samples

Formalin-fixed, paraffin-embedded tissue samples from 72 patients with primary squamous cell laryngeal carcinoma that underwent total laryngectomy (plus, neck dissection, radiotherapy, and chemotherapy were appropriate) from 1994 to 2007 were obtained from the Department of Pathology, University Hospital of Patras. Cases were revised by expert pathologists (Helen Papadaki and Vasiliki Bravou). Clinical information and survival data were obtained from the records of the Department of Otolaryngology, Head and Neck Surgery, University Hospital of Patras. Two of the cases were women, and 70 were men. Ages ranged from 28 to 89 years old (average = 61.3, median = 61). The primary tumor was glottic in 43 cases, supraglottic in 27 cases, and subglottic in 2 cases. According to the WHO classification of tumors, 13 (18.1%) were classified as well differentiated (grade I), 49 (68.1%) as moderately differentiated (grade II), and 10 (13.9%) as poorly differentiated (grade III) [32]. One case (1.4%) was stage I, 4 cases (5.6%) were stage II, 25 cases (34.7%) were stage III, and 42 cases (58.3%) were stage IV according to the TNM Classification of Malignant Tumours, 7th edition [33]. Cervical lymph nodes (N) were positive in 19 cases (26.4%) while 53 (73.6%) cases had no cervical lymph node metastases. All patients were followed up at least for 5 years. The study was performed in accordance with the Helsinki Declarations and the institutional ethical guidelines.

### 2.2. Immunohistochemistry

Immunohistochemistry was performed using a peroxidase-based polymer as previously described [27]. Primary antibodies used were rabbit polyclonal anti-nonphosphorylated (active) cofilin-1 antibody (PA1-14111, Thermo Scientific, Waltham, MA, USA; 1 : 200), anti-N-WASP antibody (sc-20770, Santa Cruz Biotechnology Inc., Santa Cruz, CA, USA; 1 : 200), anti-PARVA (*α*-parvin) antibody (HPA005964, Sigma-Aldrich, St. Luis, USA; 1 : 200), anti-*β*-parvin antibody (sc-134832, Santa Cruz Biotechnology, CA, USA; 1 : 400), and mouse monoclonal anti-PINCH-1 (MABT162, EMD Millipore, Billerica, MA, USA; 1 : 80). Bound primary antibodies were detected with the Envision™ detection kit (K5007, DAKO, Hamburg, Germany) and diaminobenzidine (DAB) as the chromogen according to the user's manual. Both positive (colorectal carcinomas) and negative controls (rabbit immunoglobulin fraction, DAKO, REFX0936) were used.

### 2.3. Immunohistochemical Evaluation

All slides were examined blinded to the case by expert pathologists (Vasiliki Bravou and Helen Papadaki). Cytoplasmic and nuclear expression was evaluated separately. Immunoreactivity was scored on a scale of 0–3 according to the intensity of staining and percentage of positive cells as previously described [27]. In short, staining intensity was graded as 0 (negative), 1 (weak), 2 (moderate), and 3 (strong). The percentage of positive cells was scored as 0 (<1%), 1 (1–25%), 2 (26–50%), 3 (51–75%), and 4 (76–100%). These two scores were multiplied resulting in immunoreactivity (IR) score with values from 0 to 12 [27]. Cases with IR score = 0 were considered negative, and cases with IR score > 0 were considered positive. For statistical purposes, cases with IR score = 0–4 were considered demonstrating low expression, while cases with IR score = 6–12 were considered demonstrating high expression of the examined protein.

### 2.4. Statistical Analysis

Statistical analysis was performed with the IBM® SPSS® for Windows, v24 (SPSS Inc., Chicago, IL, USA). Differences in the protein expression levels between groups of clinicopathological parameters were examined with the nonparametric Kruskal-Wallis and Mann–Whitney *U* tests. The correlation between the expressions of the proteins was examined using the Spearman rank-order correlation test. Overall survival (OS) was defined as the interval from the date of surgery to the death from any cause, while disease-free survival (DFS) was defined as the interval from the date of surgery to the recurrence. Overall and disease-free survivals were analyzed using the Kaplan-Meier method, and differences between subgroups were compared using the log-rank test. Cox proportional hazard univariate and multivariate analysis was performed to identify predictors of survival. Only factors that showed significance by univariate analysis were included in multivariate analysis. *p* values < 0.05 were considered statistically significant.

## 3. Results

### 3.1. Increased Expression of Actin-Binding Proteins Cofilin and N-WASP in Human Laryngeal Carcinoma

**Figure 1:**
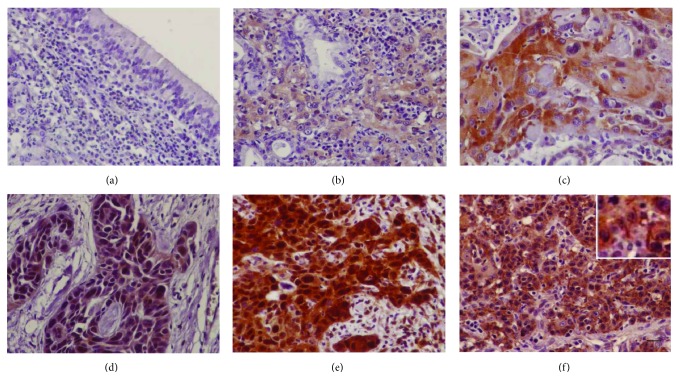
Cofilin immunohistochemical expression in human laryngeal carcinoma. (a) Negative cofilin expression in the adjacent nonneoplastic epithelium. (b) A case of laryngeal carcinoma with low cofilin expression. Representative cases of laryngeal carcinomas with high cytoplasmic (c), nuclear (d), or cytoplasmic and nuclear cofilin expression (e). (f) A case of laryngeal carcinoma with cofilin rods. Bar corresponds to 50 *μ*m (×400).

Immunoreactivity for nonphosphorylated (active) cofilin in adjacent nonneoplastic laryngeal epithelium was negative or weakly nuclear (Figure 1(a)). In carcinomas, positive immunohistochemical expression of cofilin was found in 62/72 (86.1%) cases. Immunoreactivity for cofilin was observed in the cytoplasm and/or the nucleus of cancer cells (Figure 1). Cytoplasmic expression of cofilin was found in 54/72 (75%) cases, while nuclear expression was found in 41/72 (56.9%) cases. Occasionally, in 5/72 (6.9%) cases, staining for cofilin was rod-like (Figure 1(f)). There was no statistically significant association of cofilin with any of the clinical and pathological parameters under evaluation (age, tumor location, grade, nodal metastases, or stage) (Table 1).

**Figure 2:**
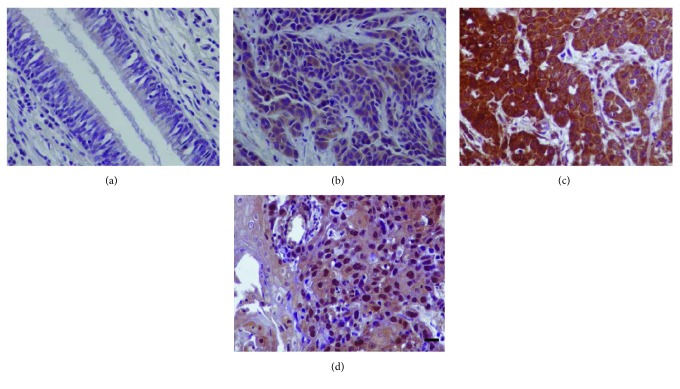
N-WASP immunohistochemical expression in human laryngeal carcinoma. (a) Negative N-WASP expression in the adjacent nonneoplastic epithelium. (b) A case of laryngeal carcinoma with low N-WASP expression. (c) Representative case of laryngeal carcinoma with high cytoplasmic N-WASP expression. (d) Nuclear expression of N-WASP in a case of laryngeal carcinoma. Bar corresponds to 50 *μ*m (×400).

Adjacent nonneoplastic laryngeal epithelium showed negative or weak staining for N-WASP, while positive immunohistochemical expression of N-WASP was found in 67/72 (93.1%) cases. Expression of N-WASP was mainly cytoplasmic while nuclear staining was observed in 7/72 (9.7%) cases (Figure 2). Expression of cytoplasmic N-WASP significantly differed among grade groups with high grade tumors showing lower expression of N-WASP (*p* = 0.032). No other statistical significant association of N-WASP with the clinical and pathological parameters under evaluation was found (Table 1). Also, nuclear N-WASP showed no significant associations in our samples.

Survival analysis with the Kaplan-Meier and log-rank test showed no significant association of cofilin or N-WASP expression with OS or DFS.

### 3.2. Focal Adhesion Proteins *α*- and *β*-Parvin Are Overexpressed in Human Laryngeal Cancer

**Figure 3:**
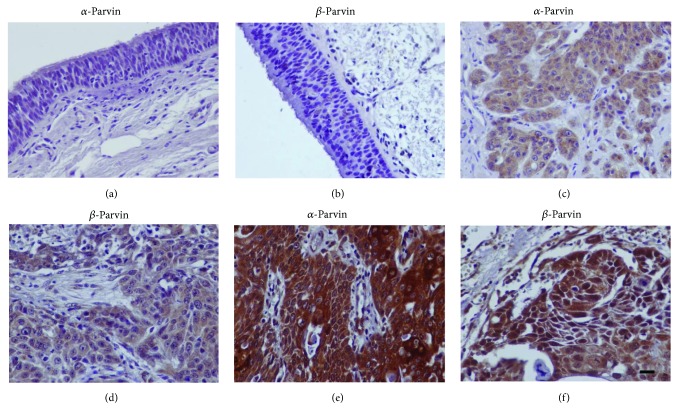
Expression of parvins by immunohistochemistry in human laryngeal carcinoma. (a, b) Negative expression in the adjacent nonneoplastic epithelium. (c, d) Representative cases of laryngeal carcinomas with low expression of parvins. (e, f) Representative cases of laryngeal carcinomas with high expression of *α*-parvin (e, cytoplasmic) and *β*-parvin (f, cytoplasmic and nuclear). Bar corresponds to 50 *μ*m (×400).

Epithelial cells of adjacent nonneoplastic tissue demonstrated negative or very weak immunoreactivity for *α*- and *β*-parvin (Figures 3(a) and 3(b)). Positive expression of *α*- and *β*-parvin in tumor cells was noted in 60/72 (83.3%) and 68/72 (94.4%) cases of laryngeal squamous carcinoma, respectively. Immunostaining of parvins was mainly cytoplasmic while a few cases (6/72, 8.3%) showed cytoplasmic and nuclear staining for *β*-parvin (Figure 3). No statistically significant association of *α*- or *β*-parvin expression with clinicopathological parameters was found (Table 2). Survival analysis with the Kaplan-Meier and log-rank test showed no significant association of parvin expression with patient survival. Immunohistochemical expression of focal adhesion proteins *α*- and *β*-parvin significantly and positively correlated with cytoplasmic expression of cofilin (*p* = 0.034 and *r* = 0.251 for *α*-parvin; *p* = 0.011 and *r* = 0.298 for *β*-parvin), and also, *β*-parvin significantly correlated with nuclear cofilin expression (*p* = 0.009, *r* = 0.304).

### 3.3. Expression of PINCH1 Is a Poor Prognostic Factor in Human Laryngeal Cancer

**Figure 4:**
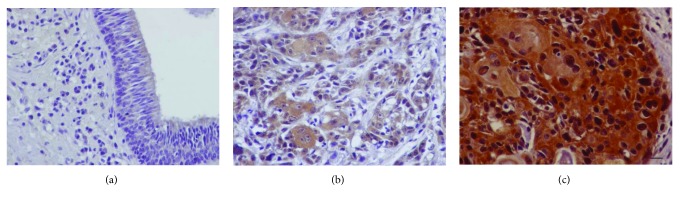
PINCH1 immunohistochemical expression in human laryngeal carcinoma. (a) Negative expression in the adjacent nonneoplastic epithelium. (b) A case of laryngeal carcinoma with low PINCH1 expression. (c) Representative case of laryngeal carcinoma with high PINCH1 expression (cytoplasmic and nuclear). Bar correspond to 50 *μ*m (×400).

**Figure 5:**
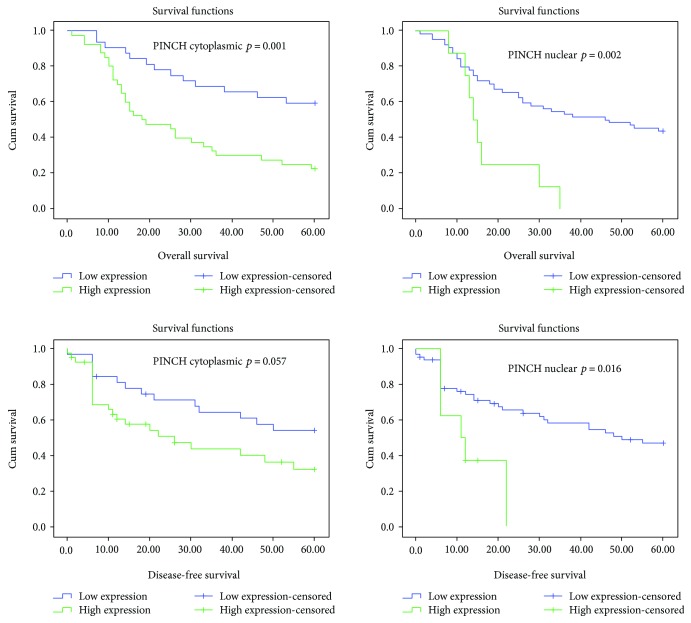
Kaplan-Mayer Survival plots. Overall survival and disease-free survival estimates according to cytoplasmic and nuclear PINCH1 expression. Log-rank test; *p* values < 0.05 are considered statistically significant.

PINCH1 expression in the adjacent nonneoplastic epithelium was negative or very weak, while cytoplasmic and nuclear expression of PINCH1 in tumor cells was detected in 69/72 (95.8%) and 22/72 (30.6%) of cases, respectively (Figure 4). Cytoplasmic expression of PINCH1 differed significantly among groups, and it was higher in high grade (*p* = 0.003), lymph node-positive (*p* = 0.004), and high stage (*p* = 0.001 for stage IV versus stage III) disease. Nuclear expression of PINCH1 was also significantly associated with high tumor grade (*p* < 0.001) (Table 3). No other significant difference was noted in the nuclear expression of PINCH1 among groups of clinicopathological parameters. The Kaplan-Mayer analysis showed a statistically significant association of high cytoplasmic and nuclear PINCH1 expression with reduced OS (28.6 versus 45.4 for low and log-rank *p* = 0.001 for cytoplasmic; 17.8 versus 38.4 for low and *p* = 0.002 for nuclear). There was also a significant association of high nuclear PINCH1 with reduced DFS (13.3 versus 38.5 for low and log-rank *p* = 0.016) (Figure 5). Univariate analysis showed that grade, N (lymph node status), and PINCH1 expression are significant predictors of poor overall (both nuclear and cytoplasmic PINCH1) and disease-free survivals (only nuclear PINCH1) (Table 4). Moreover, in multivariate analysis, high cytoplasmic PINCH1 expression was shown to be an independent predictor of poor OS (Table 5). In addition, a strong and positive correlation was found between the cytoplasmic expression of *β*-parvin and the cytoplasmic expression of PINCH1 (*p* = 0.027, *r* = 0.261).

## 4. Discussion

Reorganization of the actin cytoskeleton and focal adhesion signaling are fundamental to cancer cell invasion and metastasis [2, 21]. Actin-binding proteins cofilin and N-WASP and the focal adhesion proteins *α*- and *β*-parvin and PINCH are critically involved in the regulation of actin cytoskeleton dynamics and cell adhesion, survival, and migration, and several studies implicate their altered expression in cancer progression [2–15, 21, 22, 24–31]. Here, we originally demonstrate their overexpression in human laryngeal carcinoma and we further show that high expression of PINCH1 is an independent poor prognostic factor.

We showed that active cofilin is overexpressed in laryngeal squamous cell carcinoma suggesting a possible role for cofilin in laryngeal carcinogenesis. In agreement, cofilin is involved in the reorganization of actin cytoskeleton leading to the formation of protrusive structures that enhance motility associated with cancer cell invasion and metastasis [2–6, 34]. Overexpression of cofilin is a feature of cells exhibiting highly invasive and malignant phenotypes [6]. Its expression is elevated in a variety of human cancers, and it is frequently associated with malignant progression [7–10, 34].

Cofilin immunoreactivity in our samples was cytoplasmic and/or nuclear. The cytoplasmic localization of cofilin agrees with its role in the regulation of the actin cytoskeleton [2–5]. In addition, a nuclear localizing signal has been demonstrated in the cofilin sequence and important nuclear functions such as the transport of G-actin into the nucleus to regulate chromatin remodeling and gene transcription and regulation of nuclear architecture have been attributed to cofilin in previous studies [4, 5, 35–39]. In addition, some of our cases showed cytoplasmic and nuclear cofilin rods in tumor cells. Consistently, it has been demonstrated that cofilin-actin accumulations in rod-like patterns form in response to cellular stress and it would be interesting to further explore the significance of nuclear cofilin expression and cofilin rods in human cancer [40].

Many studies support that overexpression of cofilin in human cancer is related to adverse prognostic factors and aggressive biological behavior. Specifically, increased expression of cofilin associated with increased invasion, lymph node metastases, advanced stage, and poor prognosis in several types of cancer [5, 7–10, 36]. Moreover, several in vitro data show increased cell migration, invasion, and metastasis in cells overexpressing cofilin [5, 34]. However, we found no significant association of cofilin expression with tumor grade, nodal status, stage, and patient survival suggesting that the expression of cofilin in laryngeal cancer does not serve as a prognostic indicator. Nevertheless, this finding needs confirmation with studies that include a larger number of cases (especially early stage) and future research will further clarify the prognostic significance of cofilin overexpression in laryngeal cancer.

N-WASP was also overexpressed in our cohort of tumors suggesting a possible tumorigenic role for N-WASP in laryngeal carcinoma, consistent with reports in other malignancies [9, 13–15]. These findings are in agreement with the known role of N-WASP in the regulation of actin filament turnover and the formation of protrusive structures promoting cell migration and invasion [6, 11, 12]. High N-WASP expression in several types of cancer including squamous oesophageal carcinoma, pancreatic ductal adenocarcinoma, and hepatocellular carcinoma showed correlation with advanced stage, metastases, and reduced survival rates, suggesting that N-WASP promotes cancer progression and aggressive tumor behavior [9, 14, 15]. However, we surprisingly found that aggressive high grade tumors show lower expression of N-WASP. It should be noted though that there are studies supporting a different role for N-WASP in cancer as low expression of N-WASP associated with metastases and poor prognosis in breast cancer and induction of N-WASP expression reduced breast cancer cell invasiveness [41]. N-WASP also exerts functions in maintaining cell junctions and regulating gene expression [42, 43]. Consistent with studies showing that N-WASP localizes in the nucleus and in nuclear actin complexes, regulating gene transcription, we found nuclear localization of N-WASP in few of our cases [36, 39, 43]. Nevertheless, we found no significant correlations of N-WASP expression with survival suggesting that further evaluation of the prognostic role of N-WASP in human laryngeal cancer is required.

In addition, we demonstrated that focal adhesion proteins *α*- and *β*-parvin are overexpressed in our cohort of tumors although no significant association with adverse prognostic factors was confirmed. Parvins localize at focal adhesions with an important role in integrin signaling and in the regulation of cell adhesion, cell migration, proliferation, and survival [16–18, 21–23]. While *α*-parvin has been shown to promote cancer progression in colorectal, breast, lung, and hepatocellular carcinomas, a controversial role of *β*-parvin expression in cancer has been observed in various studies [26–30, 44–46]. Studies in breast and urothelial carcinomas favor a tumor suppressive role of *β*-parvin, whereas increased *β*-parvin expression has been demonstrated in colorectal cancer, tongue cancer, and chondrosarcomas, indicating that *β*-parvin functions in human cancer may depend on the tissue context [26, 27, 30, 44–46]. Our results are consistent with a possible tumorigenic role for both parvins in squamous laryngeal carcinomas. However, further evaluation of their significance in laryngeal cancer in larger cohorts of tumors is needed.

Interestingly, *α*- and *β*-parvin expression correlated with cofilin expression in our samples. Consistently, parvins interact with guanine nucleotide exchange factors (GEFs) and GTPase-activating proteins (GAPs) to promote integrin-induced Rho family signaling to the cytoskeleton and regulate lamellipodia formation and cell migration [47–50]. Specifically, *β*-parvin binds to the GEF factor alpha and beta PIX, which in turn is important for PAK activation and Rho signaling to the actin cytoskeleton, a process involving the LIMK/cofilin pathway [47–49, 51, 52]. *α*-Parvin also binds to the serine/threonine kinase testicular protein kinase 1 (TESK1) a ubiquitously expressed protein reported to regulate actin cytoskeleton dynamics via phosphorylation of cofilin [53, 54]. Other studies also suggest that the ILK/*β*-parvin/cofilin pathway mediates invasiveness and metastatic behavior of cancer cells [55].

We also showed for the first time that the focal adhesion protein PINCH1 is overexpressed in human laryngeal carcinoma. Apart from its cytoplasmic localization that is consistent with the function in focal adhesions, PINCH1, as well as *β*-parvin, also showed nuclear localization in some of our samples. It is interesting that ILK, their binding partner, localizes at centrosomes and mitotic spindles and regulates mitosis [56]. Both PINCH and *β*-parvin have a nuclear localization signal, and although their nuclear functions are yet unknown, an interesting hypothesis that needs to be explored is that they also interact with microtubules, similarly to their partner ILK, regulating mitosis [17, 56, 57]. Importantly, we demonstrated that high PINCH1 expression correlated with aggressive disease and adverse prognosis. Consistent with a tumor-promoting role of PINCH, it has been shown that PINCH interacts at focal adhesions sites with parvins, ILK, Nck1, Rsu-1, and several other partners to promote cell spreading, migration, and apoptosis resistance, features important for tumor cells [19–21, 24, 25]. In further agreement, increased PINCH expression has been demonstrated in tumor-associated stroma at the invasive front in several types of cancer and also high expression of PINCH in esophageal squamous carcinoma correlated with nodal metastases [24, 25, 58]. Also, increased expression of PINCH1 has been reported in colorectal carcinoma compared with a normal colon and high PINCH expression at the invasive front of colorectal cancer as well as in mucinous colorectal adenocarcinoma associated with poor survival [59–64]. Increased PINCH expression in cancer has been also associated with therapy resistance [59, 63, 64].

In further support to the implication of parvins and PINCH1 in human laryngeal cancer, we have previously shown that ILK, their binding partner, is overexpressed in laryngeal cancer [65]. As already outlined, parvins and PINCH interact with ILK in cells to form a ternary IPP complex [16, 18–21]. Noteworthy, expression of the individual members of the IPP complex depends on its formation [20]. IPP complex formation has been shown to protect its members from degradation [20]. Also, knockdown of ILK has been shown to reduce levels of parvins in intestinal epithelial cells and expression of *α*- and *β*-parvin in human colorectal cancer positively correlated with levels of ILK [27, 66]. As our previous study of ILK in laryngeal cancer involved a different tumor cohort, it would be interesting for the reasons stated above to further evaluate ILK levels in this cohort of tumors and examine associations and colocalization of ILK, parvins, and PINCH1 [65]. Different ILK expression levels or preferential formation of an IPP complex containing *β*-parvin instead of a-parvin in some tumors may for example account for the higher positivity ratio of *β*-parvin, observed in laryngeal tumors, compared to *α*-parvin.

In conclusion, our study suggests that overexpression of actin-binding proteins cofilin, N-WASP, and focal adhesion proteins *α*- and *β*-parvin and PINCH1 is implicated in human laryngeal carcinogenesis. Importantly, we provide novel evidence that high PINCH1 expression is an independent adverse prognostic indicator in human laryngeal cancer.

## Figures and Tables

**Table 1:** Expression of actin-binding proteins cofilin and N-WASP in human laryngeal cancer in relation to clinical and pathological parameters.

		Cofilin cytoplasmic expression				*p* value	Cofilin nuclear expression				*p* value	N-WASP expression				*p* value
		Low (*n*, %)		High (*n*, %)			Low (*n*, %)		High (*n*, %)			Low (*n*, %)		High (*n*, %)		
Age	≤60	18	51.4%	17	48.6%	0.825	28	80.0%	7	20.0%	0.155	14	40.0%	21	60.0%	0.782
	>60	20	54.1%	17	45.9%		24	64.9%	13	35.1%		16	43.2%	21	56.8%	

Location	Glottis	21	48.8%	22	51.2%	0.698	32	74.4%	11	25.6%	0.729	15	34.9%	28	65.1%	0.115
	Supraglottis	16	59.3%	11	40.7%		19	70.4%	8	29.6%		15	55.6%	12	44.4%	
	Subglottis	1	50.0%	1	50.0%		1	50.0%	1	50.0%		0	0.0%	2	100.0%	

Grade	Grade I	7	53.8%	6	46.2%	0.981	10	76.9%	3	23.1%	0.634	2	15.4%	11	84.6%	**0.031**
	Grade II	26	53.1%	23	46.9%		36	73.5%	13	26.5%		21	42.9%	28	57.1%	
	Grade III	5	50.0%	5	50.0%		6	60.0%	4	40.0%		7	70.0%	3	30.0%	

N	No	28	52.8%	25	47.2%	0.988	36	67.9%	17	32.1%	0.177	21	39.6%	32	60.4%	0.560
	N1–3	10	52.6%	9	47.4%		16	84.2%	3	15.8%		9	47.4%	10	52.6%	

Stage	I	1	100.0%	0	0.0%		1	100.0%	0	0.0%		0	0.0%	1	100.0%	
	II	3	75.0%	1	25.0%		3	75.0%	1	25.0%		3	75.0%	1	25.0%	
	III	14	56.0%	11	44.0%	0.510	18	72.0%	7	28.0%	0.960	13	52.0%	12	48.0%	0.135
	IV	20	47.6%	22	52.4%		30	71.4%	12	28.6%		14	33.3%	28	66.7%	

Statistical analyses were performed by nonparametric tests, and *p* < 0.05 was considered statistically significant. Lymph node (N) metastasis and stage were determined based on TNM, 7th edition [33].

**Table 2:** Expression of focal adhesion proteins *α*- and *β*-parvin in human laryngeal cancer in relation to clinical and pathological parameters.

		*α*-Parvin				*p* value	*β*-Parvin				*p* value
		Low (*n*, %)		High (*n*, %)			Low (*n*, %)		High (*n*, %)		
Age	≤60	12	34.3%	23	65.7%	0.142	16	45.7%	19	54.3%	0.251
	>60	7	18.9%	30	81.1%		12	32.4%	25	67.6%	

Location	Glottis	11	25.6%	32	74.4%	0.649	15	34.9%	28	65.1%	0.286
	Supraglottis	8	29.6%	19	70.4%		13	48.1%	14	51.9%	
	Subglottis	0	0.0%	2	100.0%		0	0.0%	2	100.0%	

Grade	Grade I	2	15.4%	11	84.6%	0.484	5	38.5%	8	61.5%	0.997
	Grade II	15	30.6%	34	69.4%		19	38.8%	30	61.2%	
	Grade III	2	20.0%	8	80.0%		4	40.0%	6	60.0%	

N	No	14	26.4%	39	73.6%	0.993	19	35.8%	34	64.2%	0.380
	N1–3	5	26.3%	14	73.7%		9	47.4%	10	52.6%	

Stage	I	0	0.0%	1	100.0%		0	0.0%	1	100.0%	
	II	1	25.0%	3	75.0%		3	75.0%	1	25.0%	
	III	6	24.0%	19	76.0%	0.685	11	44.0%	14	56.0%	0.386
	IV	12	28.6%	30	71.4%		14	33.3%	28	66.7%	

Statistical analyses were performed by nonparametric tests, and *p* < 0.05 was considered statistically significant. Lymph node (N) metastasis and stage were determined based on TNM, 7th edition [33].

**Table 3:** Expression of PINCH1 in human laryngeal cancer in relation to clinical and pathological parameters.

		PINCH1 cytoplasmic				*p* value	PINCH1 nuclear				*p* value
		Low (*n*, %)		High (*n*, %)			Low (*n*, %)		High (*n*, %)		
Age	≤60	20	57.1%	15	42.9%	**0.036**	32	91.4%	3	8.6%	0.508
	>60	12	32.4%	25	67.6%		32	86.5%	5	13.5%	

Location	Glottis	18	41.9%	25	58.1%	0.866	38	88.4%	5	11.6%	0.879
	Supraglottis	13	48.1%	14	51.9%		24	88.9%	3	11.1%	
	Subglottis	1	50.0%	1	50.0%		2	100.0%	0	0.0%	

Grade	Grade I	11	84.6%	2	15.4%	**0.003**	13	100.0%	0	0.0%	**<0.001**
	Grade II	19	38.8%	30	61.2%		47	95.9%	2	4.1%	
	Grade III	2	20.0%	8	80.0%		4	40.0%	6	60.0%	

N	No	29	54.7%	24	45.3%	**0.004**	48	90.6%	5	9.4%	0.453
	N1–3	3	15.8%	16	84.2%		16	84.2%	3	15.8%	

Stage	I	1	100.0%	0	0.0%		1	100.0%	0	0.0%	
	II	3	75.0%	1	25.0%		4	100.0%	0	0.0%	
	III	17	68.0%	8	32.0%	**0.001**	24	96.0%	1	4.0%	0.125
	IV	11	26.2%	31	73.8%		35	83.3%	7	16.7%	

Statistical analyses were performed by nonparametric tests, and *p* < 0.05 was considered statistically significant. Lymph node (N) metastasis and stage were determined based on TNM, 7th edition [33].

**Table 4:** Univariate Cox regression analysis for overall and disease-free survival.

	Univariate analysis					
	Overall survival			Disease-free survival		
	*HR*	*95% CI*	*p value*	*HR*	*95% CI*	*p value*
*Age*	1.013	0.986–1.040	0.361	1.009	0.980–1.039	0.536
*Location*			0.999			0.756
Supraglottis/glottis	1.007	0.549–1.848	0.981	1.282	0.669–2.459	0.454
Subglottis/glottis	0.000	0.000	0.979	0.000	0.000	0.981
*Grade*			**<0.001**			0.**001**
Grade II/grade I	1.094	0.476–2.513	0.832	1.039	0.421–2.563	0.934
Grade III/grade I	5.076	1.855–13.893	**0.002**	5.018	1.679–14.997	**0.004**
*N* (N0/N1–3)	3.157	1.707–5.839	**<0.001**	3.455	1.772–6.734	**<0.001**
*Stage* (I-II/III-IV)	4.753	0.654–34.552	0.124	3.953	0.541–28.895	0.176
*Cofilin cytoplasmic* Low/high	0.882	0.487–1.597	0.678	0.940	0.492–1.794	0.850
*Cofilin nuclear* Low/high	0.646	0.319–1.310	0.226	0.794	0.384–1.646	0.536
*N-WASP* Low/high	0.821	0.452–1.492	0.518	0.730	0.382–1.394	0.340
*PARVA* Low/high	0.672	0.351–1.288	0.231	0.908	0.414–1.990	0.810
*PARVB* Low/high	0.958	0.522–1.758	0.889	0.874	0.453–1684	0.687
*PINCH1 cytoplasmic* Low/high	2.856	1.487–5.485	**0.002**	1.855	0.949–3.625	0.071
*PINCH1 nuclear* Low/high	3.184	1.435–7.064	**0.004**	2.775	1.102–6.991	**0.030**

HR: hazard ratio; CI: confidence interval. Significant *p* values appear in bold.

**Table 5:** Multivariate Cox regression analysis for overall and disease-free survival.

	Multivariate analysis					
	Overall survival			Disease-free survival		
	*HR*	*95% CI*	*p value*	*HR*	*95% CI*	*p value*
*Grade*			**0.038**			**0.017**
Grade II/grade I	0.734	0.304–1.771	0.492	0.810	0.319–2.057	0.658
Grade III/grade I	3.711	0.863–15.956	0.078	4.386	1.091–17.628	**0.037**
*N* *(No/N1–3)*	2.169	1.113–4.227	**0.023**	3.076	1.508–6.274	**0.002**
*PINCH1 cytoplasmic* Low/high	2.268	1.088–4.727	**0.029**			
*PINCH1 nuclear* Low/high	0.518	0.135–1.987	0.337	0.590	0.168–2.075	0.411

HR: hazards ratio; CI: confidence interval. Significant *p* values appear in bold. Only parameters that showed a significant difference by univariate analysis were included in the multivariate analysis.
